# Editors' selection of papers from China's academic journals

**DOI:** 10.1093/nsr/nwy167

**Published:** 2019-01-03

**Authors:** Xiuling Xu

**Affiliations:** Edited

## PHYSICS

### Continuous-wave laser-enhanced pulsed-laser ablation

As thermal diffusion dominates the conventional pulsed-laser ablation process, the ablation efficiency is limited by the physical properties of samples. Minghui Hong (National University of Singapore), Lijun Yang (Harbin Institute of Technology) and co-workers reported that the irradiation of continuous-wave laser during pulsed-laser ablation can decrease the single pulse threshold fluence while increasing the pulse incubation coefficient and ablation rate of silicon and 316 L steel based on experimental and theoretical analyses, which is attributed to the weakening of the plasma shielding effect and thermal diffusion in the ablation region.

[Ding Y *et al. Sci China-Phys Mech Astron* 2019; **62**: 034211]

## PHYSICS

### Going beyond the MHz sampling rate in a distributed Brillouin optical fiber sensor

**Figure 1.**
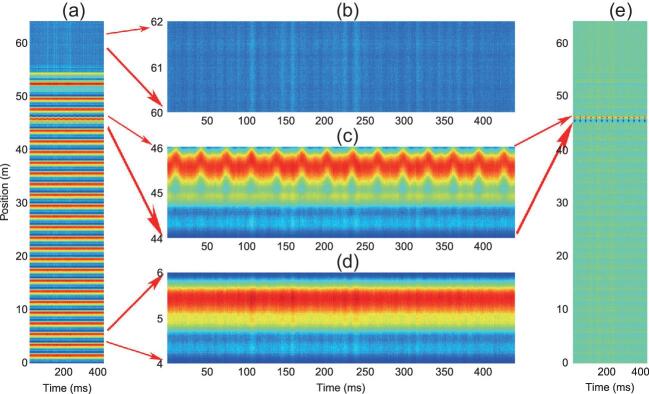
The dynamic measurement of the distributed Brillouin optical sensor using an optical chirp chain.

Ultra-fast measurement has been a big challenge for distributed Brillouin optical fiber sensors for the past two decades. A research team, led by Yongkang Dong from Harbin Institute of Technology, has proposed a technique to improve the sampling rate of the distributed measurement up to the order of ∼MHz through modulating the time-frequency mapping of the continuous probe wave to an optical chirp chain (see Fig. 1). The study shows a variety of applications ranging from monitoring the Earth's activities to motion capturing of robots and the human body.

[Zhou D *et al. Light Sci Appl* 2018; **7**: 32]

## PHYSICS

### Stabilization mechanisms of insulator surfaces

Most solid substances in nature are insulators, and their surface behaviors play a key role in many natural and industrial processes, such as biomineralization and catalysis. Compared to the success of surface science in metals and semiconductors, it has been challenging to advance the scientific understanding of insulator surfaces. Recently, Rong Yu and co-workers at Tsinghua University have solved the long-sought atomic structure of the MgAl_2_O_4_ (111) surface with aberration-corrected transmission electron microscopy, revealing an unconventional stabilization mechanism for the insulator surface, namely subsurface reconstruction and the oversaturation of surface bonds.

[Liu L *et al*. *Sci Bull* 2018; **63**: 1570–5]

## CHEMISTRY

### Combination of hydrosilylation and copolymerization for the synthesis of silicon-functionalized polyolefins

Metal-mediated olefin hydrosilylation and metal catalysed olefin polymerization are both of great academic and industrial importance. Recently, Changle Chen and co-workers from the University of Science and Technology of China combined these two techniques and reported an efficient strategy for the synthesis of silicon-functionalized polyolefins using widely available and inexpensive starting materials. In such a process, cobalt-catalysed dehydrogenative silylations of olefins with alkylsilanes afforded allylsilanes, which subsequently underwent nickel-catalysed copolymerization with ethylene to generate silicon-functionalized copolymers.

[Zhou S *et al. Sci Bull* 2018; **63**: 441–5]

## CHEMISTRY

### Paper-towel-derived N-doped porous carbon nanofibers as active materials for ORR and supercapacitors

Rational design of the cost-effective N-doped carbon materials with the hierarchical porous structures is of paramount importance for applications in electrochemical energy conversion and storage. Ying Zhu at Beihang University and co-workers demonstrated that paper towel can be considered as a carbon precursor and template for fabricating a N-doped porous carbon material with abundant pore structures and high nitrogen content. Therefore, carbon nanofibers could be employed not only as electrocatalysts for ORR, but as electrode materials for supercapacitors. The work may provide a facile method for efficiently utilizing biomass to prepare heteroatom-doped porous carbon materials as highly active bicatalysts for ORR and supercapacitors.

[Gao X *et al. Sci Bull* 2018; **63**: 621–8]

## MOLECULAR BIOLOGY & GENETICS

### PCIF1: ‘writer' of the epitranscriptomic m^6^Am mark is discovered and characterized

Gene expression can be post-transcriptionally regulated via dynamic and reversible RNA modifications. In addition to the well-known m6A, there exists a cap-specific, terminal N^6^,2′-O-dimethyladenosine (m^6^Am) modification. m^6^Am is also reversible and dynamic, hence being an emerging epitranscriptomic mark. However, its methyltransferase was unknown, significantly hindering functional and mechanistic investigation. Chengqi Yi (Peking University) and his colleagues reported the identification and characterization of the m^6^Am writer protein PCIF1. They used the classical biochemistry approach to identify the candidate methyltransferase. Both *in vitro* and *in vivo* assays reveal that PCIF1 is a terminal, cap-specific N^6^-methyltransferase. This novel discovery will shed light for future functional studies on the unique roles of this new epitranscriptomic mark.

[Sun HX *et al*. *Cell Res* 2019; **29**: 80–2]

## AGRICULTURAL SCIENCES

### Brassinosteroids regulation of cotton-fiber development

Cotton has long been cultivated worldwide for natural renewable textile fibers. The growth-promoting phytohormone brassinosteroids (BRs) play pivotal roles in fiber initiation and elongation. Recently, a comprehensive analysis of BES1 genes, which are BR signaling master transcription factors, in diploid and allotetraploid cotton species (*G. arboreum*, *G. raimondii*, *G. hirsutum* and *G. barbadense*) was reported by Fuguang Li's lab from the Institute of Cotton Research, Chinese Academy of Agricultural Sciences. This work strengthens our understanding of BRs' regulation of cotton-fiber development, and provides the foundation for future genetic improvement of fiber quality.

[Liu Z *et al. Sci China Life Sci* 2018; **61**: 1566–82]

## GEOSCIENCES

### Thermal-radiative coupling between the atmosphere and the surface for global-warming amplification

Over the past century, the Earth has experienced a steady rise in the global-mean surface temperature. A new study led by Song Yang of the Sun Yat-sen University reports that the thermal-radiative coupling between the atmosphere and the surface serves as a key amplifying mechanism that acts to amplify not only the direct warming associated with the anthropogenic radiative forcing, but also the additional warming by other positive climate feedbacks. This finding helps to clarify an outstanding issue regarding whether the changes in air temperature constitute a negative or positive feedback process.

[Hu XM *et al. Sci China Earth Sci* 2018; **61**: 1491–509]

## GEOSCIENCES

### When and how rice farming spread to south China

The southward dispersal of rice agriculture from the Yangtze River Basin has gained international attention for its role in affecting the cultural history and ecology of Island Southeast Asia. New discoveries in south China include the actual preserved rice, in connection with confident radiocarbon dating at 5000 years ago, reported by Xiaoyan Yang's team at Institute of Tibetan Plateau Research, CAS. These results fill a gap in scientific knowledge, clarifying that rice farming crossed the Wuyi and Nanling mountains at least by 5000 years ago, then proceeded in routes along rivers, eventually reaching into coastal areas by 4500–4000 years ago.

[Yang XY *et al. Sci Bull* 2018; **63**: 1495–501]

## MATERIALS SCIENCE

### Flexible wearable tactile sensor based on transparent composite dielectric

**Figure 2.**
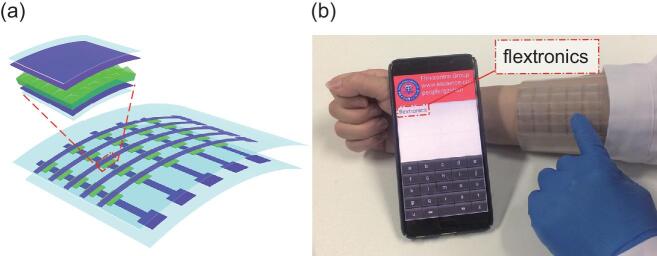
(a) Illustration of the final flexible e-skin array and an enlarged pixel with a sandwich structure. (b) Demonstration of real-time inputting word ‘flextronics' is shown in the mobile application. Adapted from *Sci China Mater* 2018; **61**: 1587–95.

The development of pressure sensors with high sensitivity, fast response and facile fabrication techniques is desirable for wearable electronics. Recently, Guozhen Shen and co-workers at the Institution of Semiconductors, CAS, reported a highly sensitive, flexible and transparent capacitive pressure sensor based on the silver nanowires/polydimethylsiloxane composite dielectric, which was then used as wearable touch keyboard systems (see Fig. 2). The fabricated pressure sensor has great potential in the applications of human–computer interaction and electronic skin.

[Shi R *et al*. *Sci China Mater* 2018; **61**: 1587–95]

## MATERIALS SCIENCE

### Achieving dispersible epitaxial heterostructures of perovskite-2D material in solution

Epitaxial heterostructures between organic–inorganic hybrid perovskites and 2D materials are promising optoelectronic materials. However, their scalable production has been restricted by the solvent incompatibility between the two material systems. Using a mixed-solvent approach, Wei Huang, Xiao Huang, Guichuan Xing and co-workers at Nanjing Tech University recently demonstrated *in situ* depositing MAPbBr_3_ nanocubes on dispersible MoS_2_ nanosheets. They further showed that, despite the large mismatch in both crystal lattice and symmetry, an unconventional epitaxial relationship between the hexagonal (001)-oriented MoS_2_ and (001)-oriented MAPbBr_3_ with a 4-fold symmetry was observed.

[Zhang ZP *et al. Sci China Mater* 2018; **62**: 43–53]

## INFORMATION SCIENCE

### Simultaneous emission-detection phenomenon for monolithic multicomponent systems

A multicomponent system on a chip with enhanced functionalities is highly demanded towards modern information-processing architecture. Yongjin Wang at Nanjing University of Posts and Telecommunications and co-workers established an on-chip optical link that integrates two identical quantum-well diodes with a suspended waveguide using all existing fabrication processes. A monolithic III-nitride multicomponent system is based on the simultaneous emission-detection phenomenon that the quantum-well diode can transmit and receive light simultaneously. The integrated system in connection with an external circuit completes full-duplex audio communication using light.

[Wang YJ *et al. Light Sci Appl* 2018; **7**: 83]

